# Endoplasmic reticulum stress and ubiquitin-proteasome system impairment in natural scrapie

**DOI:** 10.3389/fnmol.2023.1175364

**Published:** 2023-04-21

**Authors:** Jenny Lozada Ortiz, Marina Betancor, Sonia Pérez Lázaro, Rosa Bolea, Juan J. Badiola, Alicia Otero

**Affiliations:** Centro de Encefalopatías y Enfermedades Transmisibles Emergentes, Universidad de Zaragoza, IA2, IIS Aragon, Zaragoza, Spain

**Keywords:** prion, prion diseases, scrapie, endoplasmic reticulum stress, ubiquitin-proteasome system

## Abstract

Chronic accumulation of misfolded proteins such as PrP^Sc^ can alter the endoplasmic reticulum homeostasis triggering the unfolded protein response (UPR). In this pathogenic event, the molecular chaperones play an important role. Several reports in humans and animals have suggested that neurodegeneration is related to endoplasmic reticulum stress in diseases caused by the accumulation of misfolded proteins. In this study, we investigated the expression of three endoplasmic reticulum stress markers: PERK (protein kinase R-like endoplasmic reticulum kinase), BiP (binding immunoglobulin protein), and PDI (Protein Disulfide Isomerase). In addition, we evaluated the accumulation of ubiquitin as a marker for protein degradation mediated by the proteasome. These proteins were studied in brain tissues of sheep affected by scrapie in clinical and preclinical stages of the disease. Results were compared with those observed in healthy controls. Scrapie-infected sheep showed significant higher levels of PERK, BiP/Grp78 and PDI than healthy animals. As we observed before in models of spontaneous prion disease, PDI was the most altered ER stress marker between scrapie-infected and healthy sheep. Significantly increased intraneuronal and neuropil ubiquitinated deposits were observed in certain brain areas in scrapie-affected animals compared to controls. Our results suggest that the neuropathological and neuroinflammatory phenomena that develop in prion diseases cause endoplasmic reticulum stress in brain cells triggering the UPR. In addition, the significantly higher accumulation of ubiquitin aggregates in scrapie-affected animals suggests an impairment of the ubiquitin-proteasome system in natural scrapie. Therefore, these proteins may contribute as biomarkers and/or therapeutic targets for prion diseases.

## 1. Introduction

Prions are infectious agents causing fatal neurodegenerative disorders termed prion diseases or transmissible spongiform encephalopathies (TSEs) that affect both animals and humans. Examples of these disorders are Creutzfeldt-Jakob disease (CJD) and fatal familial insomnia in humans; and bovine spongiform encephalopathy in cattle (BSE), chronic wasting disease (CWD) in cervids and scrapie in sheep and goats. TSEs are characterized by the accumulation of the pathological prion protein (PrP^Sc^)in the central nervous system (CNS), an abnormal isoform of a physiological membrane glycoprotein called cellular prion protein (PrP^C^) (Prusiner, 1998).

In eukaryotic cells the endoplasmic reticulum (ER) participates in the synthesis and folding of proteins. However, it is believed that the ER plays an important role in the development of neurodegenerative diseases such as TSEs. Several studies have found that the ER function is impaired in prion diseases and that the accumulation of PrP^Sc^ perturbs ER homeostasis triggering ER stress. This situation activates a prosurvival signaling network called the unfolded protein response (UPR), causing the upregulation of ER chaperones and foldases and the reduction of protein translation (Xu and Zhu, 2012; Otero et al., 2021).

Proline-rich extension-like receptor protein kinase (PERK) is one of the major sensor proteins that activates the UPR (Wang et al., 2018). During the pathogenesis of ER stress, PERK phosphorylation reduces protein synthesis and alleviates the burden of misfolded proteins in the ER (Harding et al., 1999; Kim et al., 2022). *In vitro* and *in vivo* studies suggest that PERK phosphorylation is induced in response to prion propagation of PrP^Sc^ in neurons (Tanaka et al., 2020; Otero et al., 2021).

Another major player in ER stress is the binding immunoglobulin protein (BiP/Grp78 hereafter referred to as BiP), an ER chaperone and a primary sensor in the activation of the UPR. Misfolded proteins bind to BiP, which acts as a ER stress sensor when PERK switches BiP from its chaperone cycle (Kopp et al., 2019) and participates in the correct folding of PrP^c^ (Jin et al., 2000).

BiP overexpression was demonstrated in cells undergoing ER stress, acting as a quality control protein with high potential to reduce the accumulation of PrP aggregates (Thapa et al., 2018). Previous studies show that overexpression of BiP decreases prion propagation, thus, a reduction in the expression of this molecular chaperone accelerates prion pathogenesis in *in vitro* and *in vivo* models (Park et al., 2017). The main objective of the UPR is to assist in the refolding of these misfolded proteins, and participate in their degradation through the endoplasmic reticulum associated degradation (ERAD) mechanism, which is the principal ER quality control machinery that recognizes unfolded proteins and relocates them to the cytosol to be degraded by the ubiquitin-proteasome system to maintain homeostasis (Hwang and Qi, 2018). If these adaptive events are incapable of correcting cellular stress, cells undergo cell death by apoptosis (Hetz et al., 2011).

The role of molecular chaperones is key in the folding of PrP. One study showed that the members of the family of PDI (Protein Disulfide Isomerases), play an important role in scrapie-infected animals (Wang et al., 2012). PDI chaperones are responsible of catalyzing the formation, reduction, and isomerization of disulfide bonds of proteins (Rutkevich and Williams, 2011) and play an important role in ensuring the quality control in the PrP maturation pathway (Wang et al., 2012). PDI was overexpressed in brains of sporadic Creutzfeldt–Jakob disease patients (Yoo et al., 2002) and in brain tissues from hamsters infected with prions (Wang et al., 2012). PDI has been reported to have different roles during the prion pathogenesis (Rutkevich and Williams, 2011). At early stages of the disease, PDI overexpression has a protective activity, eliminating misfolded proteins (Rutkevich and Williams, 2011; Hetz, 2012). At the late stages, PDI activity may induce oxidative stress and triggers apoptosis (Perri et al., 2016).

The main role of the UPR is to reduce the load of misfolded proteins and maintain homeostasis in the ER. Overactivation of the UPR in cells plays a neuroprotective role in prion diseases and has been studied in models of animal and human neurodegenerative diseases (Ironside et al., 1993; Lin et al., 2013). The ubiquitin-proteasome system (UPS) is involved in the degradation of resident or abnormal proteins that accumulate in the endoplasmic reticulum. Misfolded proteins are labeled by ubiquitin molecules and degraded by the 26S proteasome complex (Lin et al., 2013). If this degradation is unsuccessful, ubiquitin aggregates will accumulate and the cell will undergo apoptosis (Hetz, 2012). Previous studies documented increased ubiquitin immunoreactivity in brain tissues from human prion diseases (Ironside et al., 1993) and in prion-infected mice (Kristiansen et al., 2007; Otero et al., 2021). Thus, UPS dysfunction contributes to the accumulation of neurotoxic proteins and promotes neurodegeneration (Zheng et al., 2016).

All the events described above are key to maintaining ER homeostasis and preventing neurodegenerative pathologies. In recent studies, scientists focusing on pathogenic mechanisms have suggested that neurodegeneration is caused by the accumulation of misfolded proteins that trigger ER stress (Perri et al., 2016; Ghemrawi and Khair, 2020). In 2021, we described the overexpression of PERK, BiP, PDI and ubiquitin in brain areas in a murine model developing spontaneous prion disease, indicating that ER and proteasome impairment occurs during pathogenesis of prion diseases (Otero et al., 2021). Therefore, the main objective of this study is to evaluate the presence of PERK, BiP and PDI proteins as potential biomarkers of ER stress in brain tissue and the presence of ubiquitinated deposits that suggest an impaired proteasome system in a natural model of prion disease: sheep naturally infected with scrapie.

## 2. Materials and methods

### 2.1. Animals and sampling

Brain samples from sheep infected with a natural prion disease and control samples were obtained from the tissue bank of Centro de Encefalopatias y Enfermedades Transmisibles Emergentes of the University of Zaragoza. Sample collection and demographic details of animals were described in a previous study (Betancor et al., 2022). Thus, we used brain samples from 21 female *Rasa Aragonesa* sheep. All sheep were 4 to 5-year-old females of the ARQ/ARQ *PRNP* genotype. 8 animals were euthanized at clinical stage, 5 animals in preclinical stage, and 8 animals were healthy controls. Euthanasia was performed by intravenous overdose of pentobarbital. Clinical signs of scrapie detected in clinical animals were pruritus, hyperesthesia, cachexia, bruxism, alopecia by continuous scratching, and hyperexcitability to external stimuli. The diagnosis of preclinical scrapie, which were obtained from a different flock, was performed by immunohistochemical analysis against PrP^Sc^ in rectal mucosa biopsies. The 8 animals from the control group were obtained from a herd in which no scrapie cases had been reported. CNS samples consisted of two replicates from nine brain areas: spinal cord (Sc), medulla oblongata (Mo), cerebellum (Cbl), hypothalamus (Ht), thalamus (T), parietal cortex (Pc), basal ganglia (BG), cortex at the level of basal ganglia (BGc), hippocampus (Hc) and frontal cortex (Fc). Brain samples were taken for histopathological and immunohistochemical analyses (fixed in 10% formalin and then embedded in paraffin) or preserved by freezing at −80°C for biomolecular analyses. For gene expression analyses, samples from frontal cortex, thalamus, hippocampus and medulla oblongata were collected in RNAlater™ solution (Thermo Fisher Scientific, Waltham, MA, USA).

### 2.2. Histological and immunohistochemical analyses

To carry out the IHQ technique we used 4-μm-thick tissue sections obtained from paraffin-embedded brain samples. Sections were incubated overnight at 56°C.

These samples were stained with hematoxylin and eosin to analyze spongiform lesions. Immunohistochemical detection of prion protein was performed using the monoclonal primary antibody L42 (1:500, R-Biopharm, Darmstadt, Germany) after formic acid treatment and proteinase K digestion, as described in a previous study (Betancor et al., 2022).

For immunohistochemical detection of the four ER stress markers we used different primary antibodies: ab79483, Abcam (Cambridge, United Kingdom) for PERK protein; ab108613, Abcam (Cambridge, United Kingdom) for BiP protein; sc-166,474, Santa Cruz Biotechnology (Dallas, Texas, USA) for PDI protein and ab7780, Abcam (Cambridge, United Kingdom) for Ubiquitin protein. After being deparaffinized, samples were boiled in a citrate buffer (pH 6.0) solution for 20 min at 96°C to retrieve the antigens. The endogenous peroxidase activity was blocked by incubation with a ready to use blocking solution (Dako Agilent, Glostrup, Denmark) for 15 min. Tissue sections were incubated overnight at 4°C with commercial monoclonal and polyclonal antibodies diluted in a precast EnVision FLEX antibody diluent (Dako, Glostrup, Denmark) (Table 1). Immunodetection was performed using an anti-rabbit Envision polymer (Dako, Glostrup, Denmark) or an anti-mouse Envision polymer (Dako, Glostrup, Denmark), for 30 min at room temperature followed by incubation with diaminobenzidine (DAB, Dako, Glostrup, Denmark) as chromogen substrate.

**Table 1:** Immunohistochemical protocols used for ER stress markers detection.

Antibody/specificity	Clone n°	Isotype	Manufacturer	Antigen retrieval	Dilution
Anti-PERK	Polyclonal ab79483	Rabbit IgG	Abcam, Cambridge, United Kingdom	Citrate pH 6.0 for 20 min at 96°C	1:250
Anti-BiP	Monoclonal ab108613	Rabbit IgG	Abcam, Cambridge, United Kingdom	Citrate pH 6.0 for 20 min at 96°C	1:500
Anti-PDI	Monoclonal F-11 (sc-166,474)	Mouse IgG	Santacruz Biotechnology, Dallas, Texas, USA.	Citrate pH 6.0 for 20 min at 96°C	1:200
Anti-Ubiquitin	Polyclonal ab7780	Rabbit IgG	Abcam, Cambridge, United Kingdom	Citrate pH 6.0 for 20 min at 96°C	1:100

PERK, Protein kinase R -like endoplasmic reticulum kinase; BiP, Binding immunoglobulin protein; PDI, Protein disulfide isomerase.

### 2.3. Evaluation and quantification

Brain sections were studied using a Zeiss Axioskop 40 optical microscope (Zeiss, Oberkochen, Germany). BiP, PERK, PDI and Ubiquitin immunostaining was blindly evaluated in the aforementioned 9 encephalic areas of the three groups of sheep described. For the analysis of the deposition of ER stress markers and ubiquitin deposition we used the same scoring scale as previous studies based on a semiquantitative assessment of the immunolabeling for the four markers (Otero et al., 2019, 2021; Betancor et al., 2022). Immunostaining was scored as: 0 (absence of immunostaining), 1 (minimal to slight immunostaining found in a reduced amount of brain cells), 2 (slight immunostaining present in cells from several areas of the evaluated tissue section), 3 (moderate immunostaining present in >50% of the cells of the tissue section), 4 (intense immunostaining observed in >50% of the cells of the tissue section and several areas of the neuropil) and 5 (widespread intense immunostaining throughout the entire section, observed in cells and the neuropil). Scoring of slides was performed blindly by two independent pathologists, and the results were averaged. Spongiosis and PrP^Sc^ scores, obtained in a previous study (Betancor et al., 2022) were used for a Spearman’s correlation test.

### 2.4. Gene expression analyses

The expression profile of the *EIF2AK3, HSPA5, P4HB* genes; encoding PERK, BiP and PDI proteins, respectively, was determined in tissue samples collected in RNAlater Solution (Thermo Fisher Scientific, Waltham, MA, USA). A total of 100 mg from the frontal cortex, thalamus, hippocampus and medulla oblongata areas were subjected to RNA extraction using a RNeasy Lipid Tissue Mini kit (QIAGEN, Venlo, Netherlands) following the manufacturer’s recommended protocol. QScript cDNA Super Mix (Quanta Bioscience™, Beverly, MA, USA) was used to obtain complementary DNA (cDNA) from a total of 1ug of RNA. The resulting cDNA was diluted 1:5 in pure water for further analyses. Primers design for *EIF2AK3, HSPA5, P4HB* was performed with the Primers3Plus program, a widely used tool for primer selection (Untergasser et al., 2012). The sequences, accession numbers, and concentrations of the used primers can be found in Table 2.

**Table 2:** Primers used for the genomic study.

Gene	Primer and probe sequences (50,30)	nM	Accession Number
*EIF2AK3*	F: AGGTCTCCGTTGCAGATTGG R: ACTCCATCACTGGGGGTGTA	300 300	XM_004005901.5
*HSPA5*	F: CCCGTGGCATAAACCCAGAT R: GGTCATGACACCTCCCACAG	200 200	XM_004005637.4
*P4HB*	F: TCAGACTCCGCAAAGCAGTT R: CAAAGTTGTTCCGGCCTTCG	300 300	XM_027974277.2
*GAPDH*	F: TCCATGACCACTTGGCATCGT R: GTCTTCTGGGTGGCAGTGA	900 900	AF_035421
*SDHA*	F: CATCCACTACATGACGGAGCA R:ATCTTGCCATCTTCAGTTCTGCTA	300 300	AY_970969

The quantitative real-time quantitative (qPCR) assays were performed using the StepONE Real-Time PCR System (Termo Fisher Scientific, Waltham, MA, USA). Amplifications were carried out using a total volume of 10 μL reaction solution containing 8 μL of SYBR^®^ Green Master Mix (Applied Biosystems, Waltham, MA, USA) and 2 μL of diluted cDNA. Each assay was performed with technical triplicates for each of the samples. Amplification conditions for quantitative real-time polimerase were 95°C for 10 min followed by 40 cycles of 95°C for 3 s and 60°C for 30 s. The expression of the housekeeping genes *GAPDH* and *SDHA* was used to normalize the results since both genes were described as reference genes in scrapie (Lyahyai et al., 2009). Finally, the relative gene expression quantification analyses were performed using the 2^−∆∆Ct^ method. Data were statistically analyzed with the one-way ANOVA, with Bonferroni *Post Hoc* multiple comparisons test using Prism 6 for Windows (GraphPad Software).

## 3. Results

### 3.1. ER stress markers are overexpressed in scrapie-infected sheep

**Figure 1:**
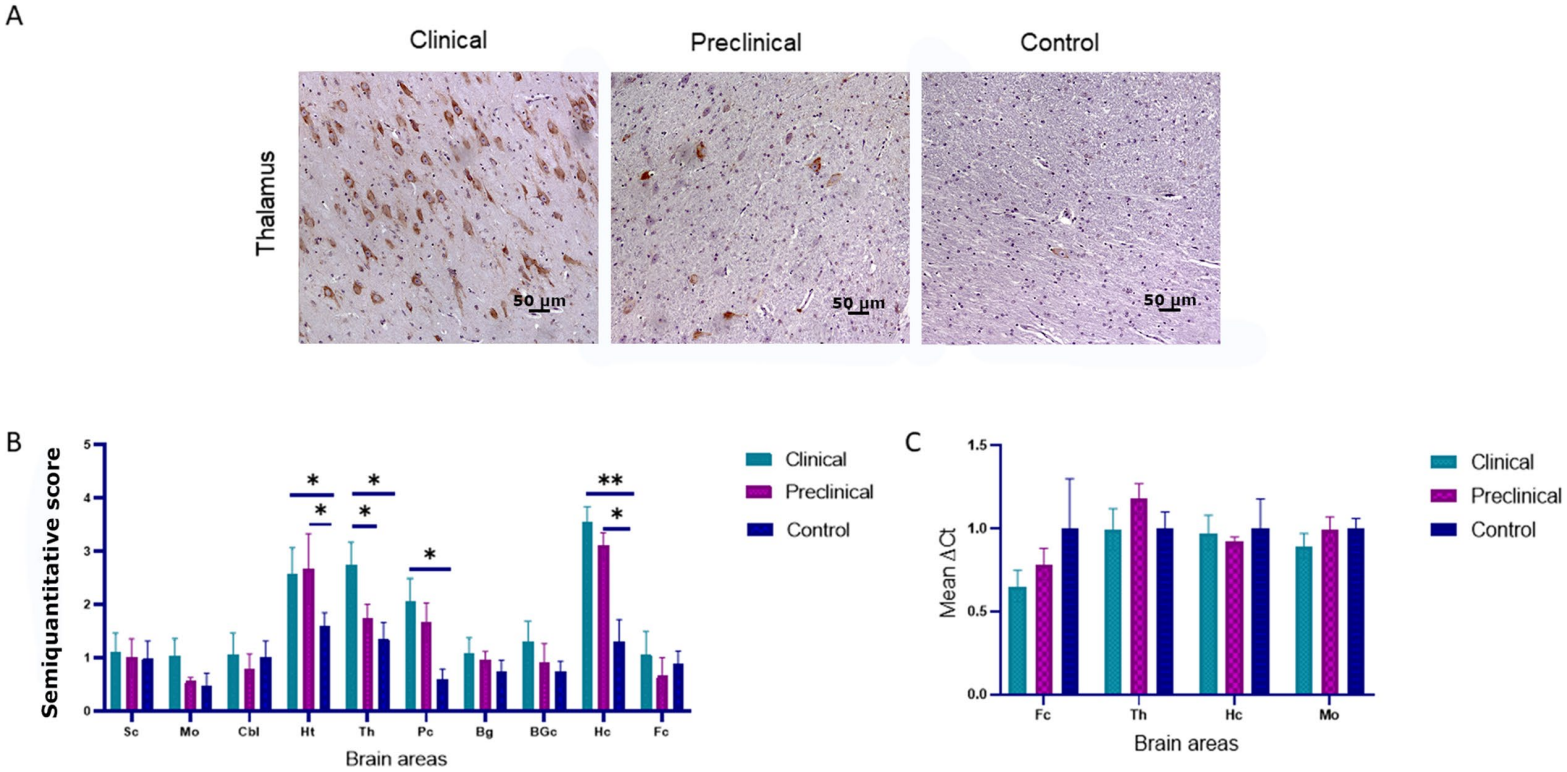
PRK-like Endoplasmic Reticulum Kinase (PERK) expression in clinical and preclinical scrapie-infected sheep and healthy sheep. **(A)** Immunohistochemical detection of PERK in thalamus. PERK-positive immunostaining was detected in cellular nuclei from clinical, preclinical and control sheep. A higher number of immunopositive cells is observed in the clinical group. **(B)** Expression levels of protein PERK in eight clinical, five preclinical and eight control animals evaluated using the following semi-quantitative scoring system a rating of 0 (lack of immunostaining) to 5 (very intense immunostaining) in nine brain areas: [spinal cord (Sc), medulla oblongata (Mo), cerebellum (Cbl), hypothalamus (Ht), thalamus (Th), parietal cortex (Pc), basal ganglia (BG), cortex at the level of basal ganglia (BGc), hippocampus (Hc) and frontal cortex (Fc)]. Clinical sheep showed the highest scores in the hippocampus, thalamus, hypothalamus and the cortex at the level of the thalamus followed by preclinical animals with similar scores in the same areas. Comparison of means was analyzed using the nonparametric Mann–Whitney U test (*p < 0.05, **p < 0.01, Mann–Whitney U test) (Figure 1B). **(C)** Gene expression of *EIF2AK3* in frontal cortex, thalamus, hippocampus and medulla oblongata of clinical, preclinical, and control sheep. *SDHA* and *GAPDH* were used as housekeeping genes. Results are expressed as the mean ± standard deviation. The expression values were determined using the 2^−∆∆Ct^ method, and differences between experimental groups were analyzed using the one-way ANOVA test, followed by the Bonferroni *post hoc* test. No significant differences were found.

PERK immunoreactivity was observed in cellular nuclei of neurons and glial cells in all sheep (clinical, preclinical and healthy controls) (Figure 1A). We observed the same pattern in a previous study using transgenic mice (Otero et al., 2021).

Comparison of PERK immunolabeling revealed significant differences between groups. The hypothalamus, thalamus, parietal cortex and hippocampus were the regions showing the most intense PERK expression in scrapie-infected sheep. Statistically significant differences in the presence of PERK positive cells were found between clinical and healthy sheep in the hippocampus (**p < 0.01), hypothalamus, thalamus and parietal cortex (*p < 0.05). Significant differences in PERK immunostaining were also observed between preclinical and healthy animals in the areas of hypothalamus and hippocampus (*p < 0.05), suggesting that the overexpression of PERK in these areas starts during the preclinical phase of the disease (Figure 1B).

The expression of the *EIF2AK3* gene, which encodes PERK protein, was analyzed by quantitative PCR in four brain areas (medulla oblongata, hippocampus, thalamus, and frontal cortex) of the three sheep groups to determine their mRNA expression levels throughout the course of the disease. Figure 1C shows the mean ∆Ct values of *EIF2AK3*. No significant differences in *EIF2AK3* expression were found between sheep groups.

**Figure 2:**
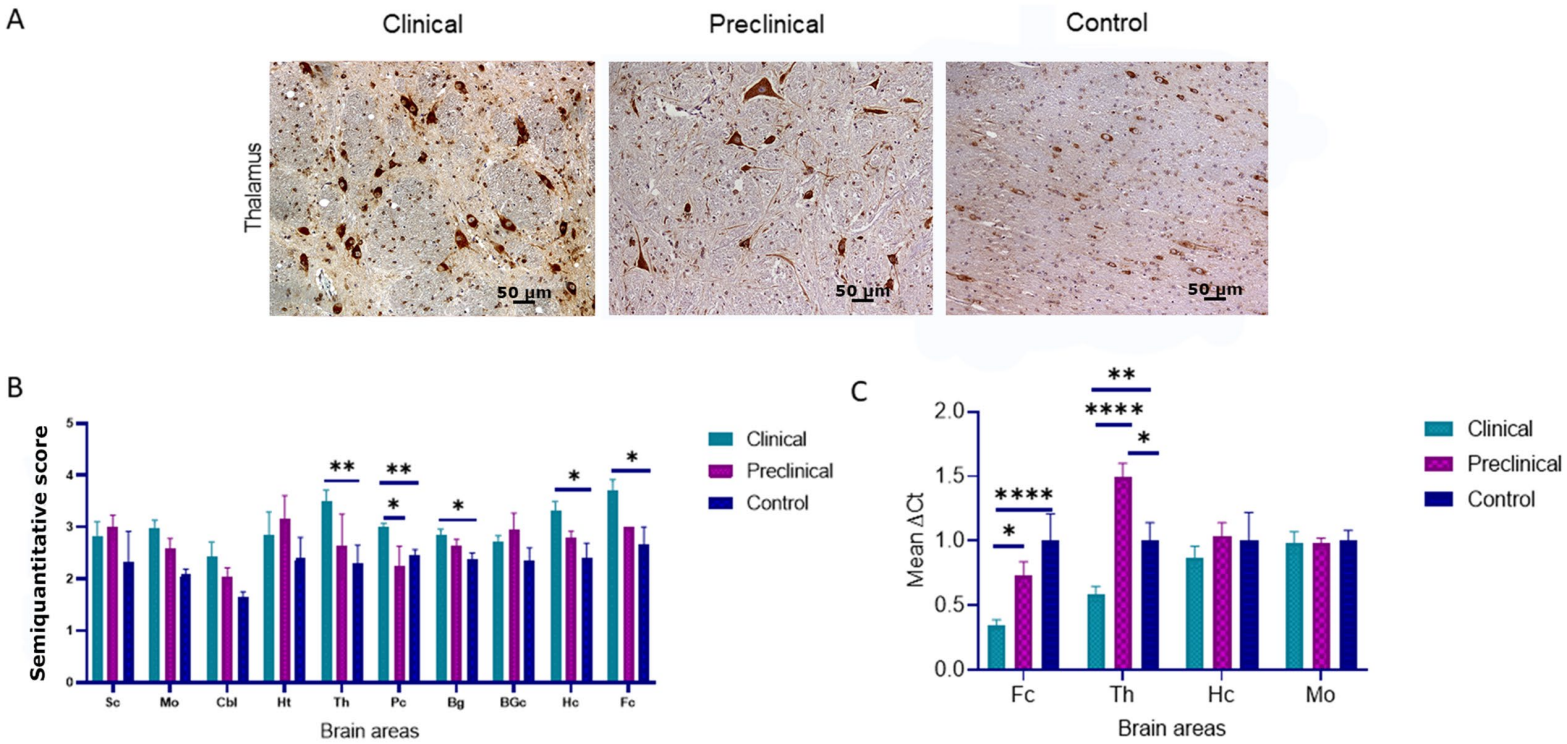
Binding immunoglobulin protein (BiP) expression in clinical and preclinical scrapie-infected sheep and control sheep. **(A)** Immunohistochemical detection of BiP in clinical, preclinical and control groups. Representative images correspond to the thalamus. Intense deposits of BiP protein were observed in the cytoplasm of neurons in clinical and preclinical sheep. **(B)** Expression levels of BiP in eight clinical, five preclinical and eight control animals were evaluated using the following semi-quantitative scoring system a rating of 0 (lack of immunostaining) to 5 (very intense immunostaining) in nine brain areas: spinal cord (Sc), medulla oblongata (Mo), cerebellum (Cbl), hypothalamus (Ht), thalamus (T), parietal cortex (Pc), basal ganglia (BG), cortex at the level of basal ganglia (BGc), hippocampus (Hc) and frontal cortex (Fc). Clinical sheep showed significantly higher deposition of BiP when compared with preclinical and control animals (*p < 0.05, **p < 0.01, Mann–Whitney U test). **(C)** mRNA expression profiles of the *HSPA5* gene in frontal cortex, thalamus, hippocampus and medulla oblongata of clinical, preclinical, and control sheep. Relative expression levels are expressed as the mean ± standard deviation. The results were normalized using the expression of *SDHA* and *GAPDH* housekeeping genes. The expression values were determined using the 2^−∆∆Ct^ method, and differences between experimental groups were assessed using the one-way ANOVA test followed by the Bonferroni *post hoc* test (*p < 0.05).

Immunohistochemical staining of BiP shows its expression as granules in the neuropil and intraneuronal deposition of the protein in all groups of sheep (Figure 2A). Statistical analysis showed that clinical sheep presented significantly higher levels of BiPin the thalamus, parietal cortex (**p < 0.01) basal ganglia, hippocampus and frontal cortex (*p < 0.05) when compared with healthy sheep. Significant differences were also observed in the parietal cortex between clinical and preclinical sheep (*p < 0.05) (Figure 2B). Quantitative PCR was performed to assess the expression of *HSPA5*, the gene that encodes the BiP protein, in the four brain areas previously used to study *EIF2AK* expression in the three study groups. Figure 2C represents the mean ∆Ct values of *HSPA5*. Clinical animals showed a downregulation of the gene compared with preclinical and control animals, in frontal cortex and thalamus. In contrast, preclinical sheep showed an upregulation of this gene in thalamus compared to control sheep.

**Figure 3:**
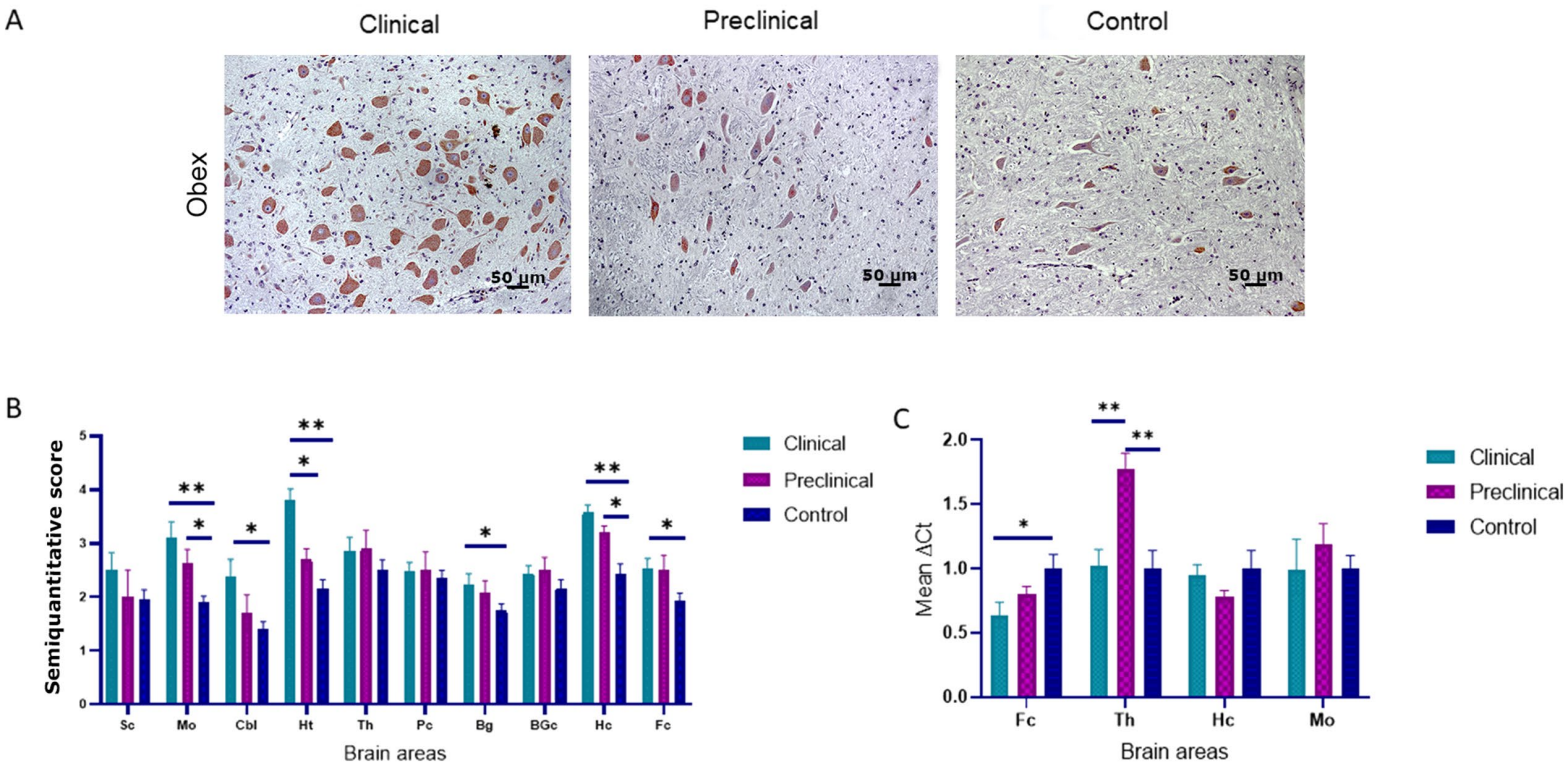
Protein disulfide isomerase (PDI) expression in clinical and preclinical scrapie-infected sheep and control sheep. **(A)** A strong intraneuronal labeling of PDI was observed in the medulla oblongata of clinical animals [pictures show the dorsal motor nucleus of the vagus nerve (DMNX)]. **(B)** PDI protein expression levels are more intense in clinical sheep when compared to controls in most brain areas. Expression levels of PDI protein in eight clinical, five preclinical and eight control animals were semi quantitatively evaluated using a scale of 0 (lack of immunostaining) to 5 (very intense immunostaining) in nine brain areas: [spinal cord (Sc), medulla oblongata (Mo), cerebellum (Cbl), hypothalamus (Ht), thalamus (Th), parietal cortex (Pc), basal ganglia (BG), cortex at the level of basal ganglia (BGc), hippocampus (Hc) and frontal cortex (Fc)]. Clinical sheep showed the highest levels of PDI in almost every brain area evaluated (*p < 0.05, **p < 0.01, Mann–Whitney U test). **(C)** Gene expression of *P4HB* in frontal cortex, thalamus, hippocampus and medulla oblongata of clinical, preclinical, and control sheep. The results were normalized using the expression of *SDHA* and *GAPDH* housekeeping genes. The expression values were determined using the 2^−∆∆Ct^ method. Mean scores between experimental groups were assessed using the one-way ANOVA test followed by the Bonferroni *post hoc* test (*p < 0.05).

PDI immunolabeling pattern was characterized by intraneuronal staining and granular deposits in the neuropil in all groups of sheep (Figure 3A). In the medulla oblongata, PDI immunostaining was found mainly in the dorsal motor nucleus of the vagus nerve (DMNX). Comparison of PDI immunolabeling revealed higher significant differences between clinical and healthy sheep in numerous brain areas (medulla oblongata, hypothalamus, hippocampus cerebellum, basal ganglia and frontal cortex). Preclinical animals showed significantly higher deposition of PDI in medulla oblongata and hippocampus compared to controls. Only in the hypothalamus we observed differences between clinical and preclinical sheep (Figure 3B).

The expression of the *P4HB* gene, encoding PDI, was analyzed by quantitative PCR in four brain areas (medulla oblongata, hippocampus, thalamus, and frontal cortex) of the three sheep groups to determine their mRNA expression levels. Figure 3C shows the mean ∆Ct values of *P4HB*. In preclinical sheep we found an upregulation of *P4HB* in thalamus compared to either clinical (*p* < 0.01) and control animals (*p* < 0.01). Clinical animals showed a downregulation in the frontal cortex region when compared to controls (*p* < 0.05).

### 3.2. The ubiquitin-proteasome system is impaired in scrapie-infected sheep

**Figure 4:**
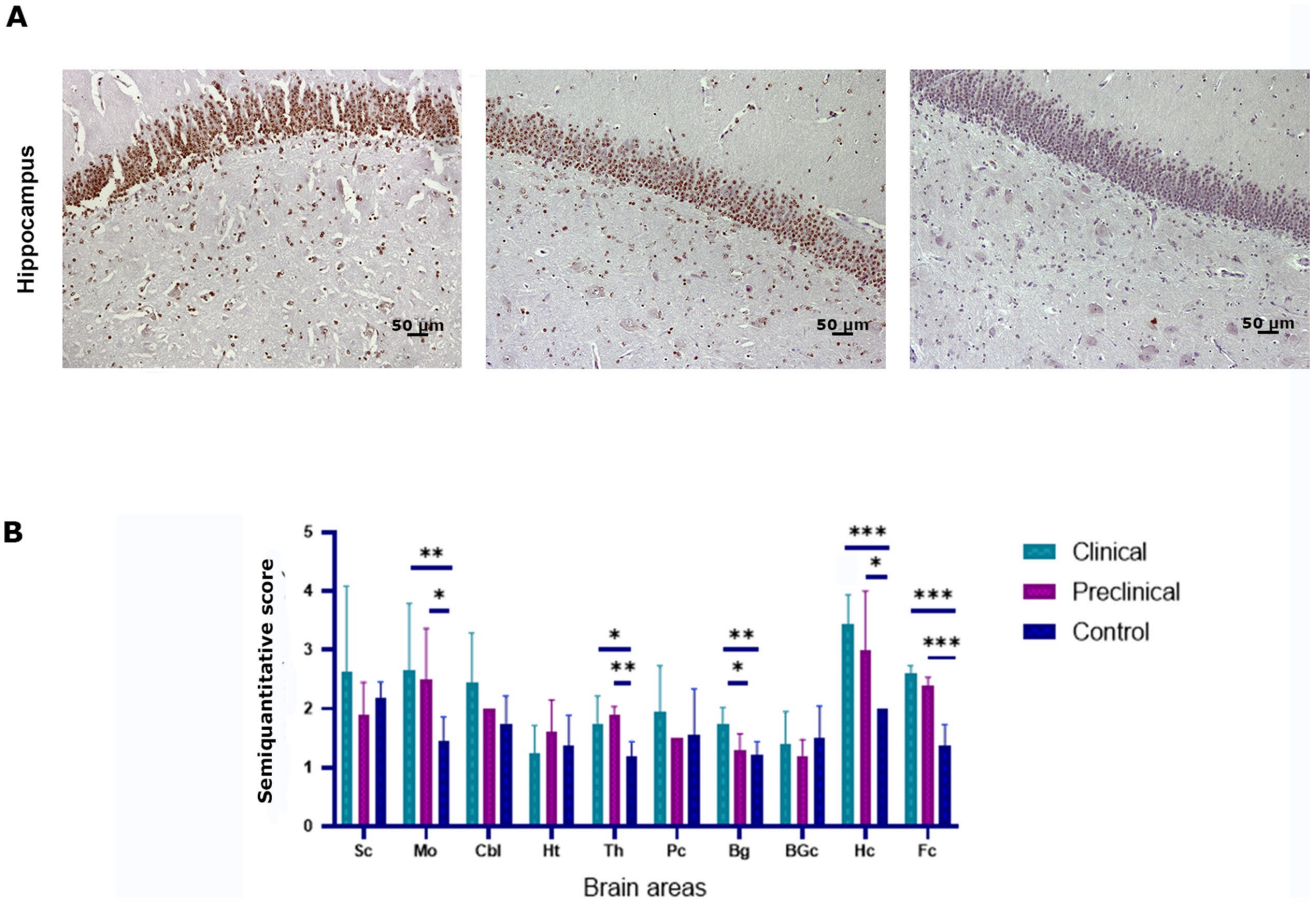
Ubiquitin accumulation in clinical and preclinical scrapie-infected sheep and healthy sheep. **(A)** Ubiquitin-protein intraneuronal aggregates are observed in the hippocampus. The CA1-CA2 regions of the hippocampus showed the strongest immunostaining for ubiquitin in clinical and preclinical sheep. **(B)** Expression levels of ubiquitin protein in eight clinical, five preclinical and eight control animals were semiquantitatively evaluated using a scale of 0 (lack of immunostaining) to 5 (very intense immunostaining) in nine brain areas: [spinal cord (Sc), medulla oblongata (Mo), cerebellum (Cbl), hypothalamus (Ht), thalamus (Th), parietal cortex (Pc), basal ganglia (BG), cortex at the level of basal ganglia (BGc), hippocampus (Hc) and frontal cortex (Fc)]. Comparison of Ubiquitin immunolabeling revealed significant differences between clinical and control sheep in several brain areas. Preclinical animals showed higher levels of ubiquitin accumulation in the frontal cortex, thalamus (**p < 0.01) medulla oblongata and hippocampus (*p < 0.05) when compared to controls. Clinical and preclinical animals showed significant differences in the basal ganglia area. Comparison of means was analyzed using the nonparametric Mann–Whitney *U* test.

To determine whether scrapie infection causes an impairment of the ubiquitin-proteasome degradation system the accumulation of ubiquitin aggregates was determined by immunohistochemistry in the brain of scrapie-infected (clinical and preclinical) and healthy sheep. Intraneuronal and neuropil ubiquitinated deposits in the form of granules were found in all groups of animals. However, scrapie infected sheep showed higher levels of ubiquitin deposition in several brain areas, such as the hippocampus (Figure 4A). Statistical analysis showed that clinical sheep had significantly higher levels of ubiquitin deposits in medulla oblongata, basal ganglia, hippocampus, frontal cortex and thalamus when compared to controls. Preclinical animals showed significant differences with controls in the same areas, except in basal ganglia. Preclinical and clinical animals showed significant differences in basal ganglia (Figure 4B).

### 3.3. Correlation between PERK, BiP, PDI, and ubiquitin proteins and neuropathological lesions

Spearman’s ρ correlation (Table 3) was calculated with the values obtained in the immunohistochemical study for ER protein stress markers, spongiform lesions and PrP^Sc^ deposition (Supplementary Figures S1, S2) to determine whether there is a possible correlation between ER stress and the impairment of the ubiquitin-proteasome system with neuropathological lesions in sheep naturally infected with scrapie. Correlations were performed using the scores obtained for each marker through the whole brain, following previous studies using this kind of analysis in neurodegenerative diseases (López-Pérez et al., 2019, 2020; Otero et al., 2021). The accumulation of ER stress markers and ubiquitin showed a significant positive correlation with spongiosis and PrP^Sc^ deposition in the brains of sheep, except for PERK, which was not correlated with spongiosis. Among these markers, PDI and ubiquitin were the proteins with the highest correlation levels with PrP^Sc^ deposition. Our results suggest that the accumulation of PDI, ubiquitin, BiP, and PERK proteins is related with the neuropathological phenomena developed in scrapie.

**Table 3:** Spearman’s correlation values between scores of endoplasmic reticulum (ER) stress, proteasome impairment markers, and prion-associated histopathological lesions.

	PERK	BiP	PDI	Ubiquitin	Spongiosis	PrP^Sc^
PERK	–	0.2079*	0.3501****	0.06418n.s	0.1557n.s.	0.2319**
BiP	0.2079*	–	0.2975***	0.2431**	0.2990**	0.2137*
PDI	0.3501****	0.2975***	–	0.1486*	0.2534**	0.3545****
Ubiquitin	0.06418n.s.	0.2431**	0.1486*	–	0.1840*	0.3090***
Spongiosis	0.1557n.s.	0.2990**	0.2534**	0.1840*	–	0.7373****
PrP^Sc^	0.2319**	0.2137*	0.3545****	0.3090***	0.7373****	–

Correlations were calculated using the complete set of data obtained in all brain areas. PERK, PKR-like endoplasmic reticulum kinase; BiP, Binding immunoglobulin protein; PrP^Sc^, Pathological prion protein; n.s., no statistically significant value. Spearman’s correlation *p < 0.05, **p < 0.01, ***p < 0.001, and ****p < 0.0001.

## 4. Discussion

In recent years, ER and UPS impairment have been suggested to be implicated in certain neurodegenerative diseases, including Parkinson, Alzheimer and prion diseases among others, which share common pathogenic mechanisms based on the accumulation of misfolded proteins (Xiang et al., 2017; Ghemrawi and Khair, 2020).

Other studies concerning ER and UPS impairment in prion and prion-like diseases have found contradictory results (Quaglio et al., 2011). In previous studies (Otero et al., 2021), we demonstrated the upregulation of PERK, BiP, PDI and ubiquitin in murine models of a spontaneous prion disease, indicating that ER and proteasome impairment are important events during the pathogenesis of prion diseases. However, as previously demonstrated, acquired, spontaneous and genetic prion diseases may not share the same pathogenesis (Quaglio et al., 2011; McKinnon et al., 2016). Moreover, previous studies on different proteins related to the pathogenesis of prion diseases have led to different results when comparing experimental and natural models of prion disease (Barrio et al., 2021; García-Martínez et al., 2022). Since there are no studies related to ER and UPS impairment on natural prion models, especially in preclinical individuals affected by a prion disease, the objective of this study was to determine whether these pathogenic markers were altered throughout the course of the disease in naturally scrapie infected sheep. In prion diseases neurons experience “ER stress” due to the accumulation of pathogenic prion protein PrP^Sc^ (Xu and Zhu, 2012). Immediately, cells trigger the UPR and the overexpression of chaperones in response to ER stress to assist refolding and maintain the cell homeostasis. If this mechanism fails, chaperones target the misfolded protein for degradation through the UPR pathway (Ciechanover and Kwon, 2017). However, prolonged ER stress in neurons leads to cell death producing neurodegeneration (Zheng et al., 2016; Ciechanover and Kwon, 2017). In addition, PrP^Sc^ aggregates cause proteasome impairment, resulting in the accumulation of PrP^Sc^ that leads to neuronal disruption and contributes to extensive neuronal loss in prion-infected mice (Kristiansen et al., 2007). In 2021, we demonstrated the up-regulation of chaperones PERK, BiP and PDI in response to ER stress in a spontaneous model of prion disease (Otero et al., 2021). Other authors have also proven that ER stress develops in brains of prion-infected mice (Hetz et al., 2003; Brown et al., 2005; Moreno et al., 2012). In this study, we investigated the accumulation of these markers of ER stress and evaluated the impairment of the proteasome activity in a natural model of prion disease.

Several authors have focused their studies on ER stress and proteasome impairment in prion diseases, but the results are limited and contradictory. PERK and BiP have been studied as markers of ER stress in brain tissues of CJD patients (Costa et al., 2010) and in spontaneous prion disease in mice (Otero et al., 2021). Other studies showed PERK activation in brain tissues from Alzheimer’s (AD) patients, and the authors suggested an important role for the UPR in the initial stages of AD neurodegeneration (Hoozemans et al., 2005, 2009).

In this study, the expression of PERK in scrapie-infected sheep was increased in several brain areas, such as the hippocampus, thalamus and hypothalamus, when compared to controls. Our results are consistent with those obtained in murine models of spontaneous prion disease (Otero et al., 2021). The upregulation of phosphorylated PERK in the hippocampal region has been described in prion-infected mice, and it has been associated with an increase in the phosphorylated form of the α-subunit of the eukaryotic translation initiation factor (eIF2α-P), which leads to neuronal loss (Moreno et al., 2012). In addition, immunohistochemical increase of PERK was described in the hippocampus in AD, especially affecting neurons in CA1 and CA2 regions (Hoozemans et al., 2005; Unterberger et al., 2006).

The upregulation of this protein was also detected in the hippocampus and hypothalamus in preclinical sheep when compared to controls. An upregulation of PERK during the preclinical phase of a spontaneous prion disease in mice was also detected in our previous study (Otero et al., 2021). These results confirm the activation of the UPR in early stages of neurodegenerative diseases (Hoozemans et al., 2009).

Spearman’s ρ correlation (Table 3) was used to determine correlations between the ER protein stress markers, spongiform lesions and PrP^Sc^ deposition. The main objective of this analysis is to know whether the expression of these proteins and the intensity of neuropathological lesions is correlated throughout the brain, independently from the area, and therefore correlations were performed for the whole brain, as previously done in other studies (López-Pérez et al., 2019, 2020; Otero et al., 2021). Although we did not find a correlation between the levels of PERK and the intensity of spongiosis, PERK expression showed a positive correlation with deposition of PrP^Sc^ (Table 3). The correlation between PrP^Sc^ deposition levels and PERK staining was also reported in an *ex vivo* study of prion-infected cortical neurons, and was associated to the early neuronal cellular response of the PERK-eukaryotic initiation factor 2 (eIF2α) (Tanaka et al., 2020). Interestingly, in this study, the authors did not find evident neurodegeneration or activation of the UPR through the PERK-eIF2α pathway, suggesting that the accumulation of prions induces ER stress in a neuron-autonomous manner (Tanaka et al., 2020). Spearman’s test showed also a positive correlation between PERK and BiP levels (Table 3), as we also observed in our previous study (Otero et al., 2021). Under normal conditions, BiP binds to the PERK stress sensor, functioning as a primary stress detector. When misfolded proteins accumulate, BiP activates the PERK pathway, and therefore the expression of these proteins is correlated (Hetz, 2012). However, as we observed before in models of spontaneous disease, PERK appears to be correlated with prion pathology in sheep with natural scrapie but not so strongly compared to the other ER stress markers. This conclusion is supported by our results in the expression of the *EIF2AK3* gene, that encodes PERK, since we did not find significant differences in *EIF2AK3* expression between prion infected and healthy sheep.

The involvement of the PERK-eukaryotic initiation factor 2 (eIF2α)-ATF4 pathway in prion diseases is still controversial. Depending on the mechanism of PERK activation, this pathway may have a proadaptive or proapoptotic role. In the proadaptive role, protein synthesis is suppressed so as not to burden the ER, through a negative feedback loop that activates the PERK-ATF4 pathway leading to the expression of several genes encoding CHOP and BiP proteins, among others, whose function is the dephosphorylation of P-eIF2α to restore the protein synthesis before neurons undergo apoptosis (Hetz et al., 2003). Recently, inhibitors of the PERK pathway have been tested in murine models, but understanding the mechanisms of PERK regulation remains a challenge (Moreno et al., 2013). Indeed, PERK inhibitors have shown neuroprotective effects in preclinical studies in mice (Moreno et al., 2013; Radford et al., 2015). Other authors agree that PERK activation could be beneficial (Hoozemans et al., 2009; Bruch et al., 2017). However, all this remains questionable since prolonged PERK activation inhibits protein synthesis and promotes neurodegeneration (Ohno, 2018). In our study, the stress sensor protein PERK shows an strong significant positive correlation with PDI, but a weak significant positive Spearman’s correlation to BiP and a non-significant correlation to ubiquitin. These findings could indicate that the molecular mechanism of PERK regulation would be mediated by PDI, being indispensable the presence of this protein to allow the oligomerization and activation of PERK (Kranz et al., 2017).

Another major player in ER stress is the ER chaperone BiP. It has been described that the expression and activity of BiP is essential for neuroprotection, to prevent protein aggregation and to regulate proper signaling of UPR (Park et al., 2017). This protective role of BiP has also been described in prion infected mice (Park et al., 2017).

There are reports revealing significant increases in the BiP chaperone expression in spontaneous prion disease (Otero et al., 2021), in *in vitro* and *in vivo* studies in prion-infected mice (Park et al., 2017), as well as in sporadic cases of CJD (Hetz et al., 2003). Also, upregulation of this protein was observed in AD models (Roller and Maddalo, 2013). In this study, we have detected significantly higher levels of BiP in several brain areas of prion infected animals, but these differences were only observed between the clinical and the control group. No significant differences in the accumulation of BiP were found between the preclinical and control groups, in contrast with our previous findings in transgenic mice (Otero et al., 2021). However, these mice developed a spontaneous prion disease, not a natural prion infection like the sheep in the present study, and, therefore, the activation of this chaperone may vary according to the different nature of the prion disease or even the infecting prion strain. Other authors have found an upregulation of BiP only in the terminal stage of murine scrapie, supporting this conclusion (Turano et al., 2002). We observed an increase in BiP protein deposition in the frontal cortex, thalamus, parietal cortex, basal ganglia and hippocampus of clinical sheep. We also analyzed the expression of the *HSPA5* gene in four areas of the brain. In the frontal cortex and thalamus, the expression of the *HSPA5* gene showed a clear downregulation in clinical animals compared with controls. In contrast, a positive regulation of the *HSPA5* (BiP) gene was reported in cortical neurons of prion-infected mice (Tanaka et al., 2020). However, in the thalamus, our preclinical animals exhibited a significant upregulation of the *HSPA5* gene. Other authors did not find an upregulation of this gene in thalamic neurons in prion-infected mice (Tanaka et al., 2020). Studies in prion disease models suggest that the thalamus displays prion deposition prior to cortex and hippocampus and is affected more severely than other brain regions (Carroll et al., 2016; Makarava et al., 2020).

Our results showed that BiP was positively correlated with PrP^Sc^ deposits and spongiosis in prion infected sheep. However, as seen with PERK, BiP is correlated to PrP^Sc^ deposition but not so strongly compared to other markers (Table 3). We, and other authors, have suggested that the PERK-eIF2α pathway may not play a crucial role in neurodegeneration in prion diseases (Unterberger et al., 2006; Otero et al., 2021). As we observed in previous studies (Otero et al., 2021), Spearman’s test confirmed a strong correlation between the expression of BiP/Grp78 and PDI. This could be due to the fact that BiP/Grp78 as master regulator of the unfolded protein response, activates the PERK-eIF2-ATF4 pathway leading to cell death or activates the IRE1 or ATF6 pathway branches as a cell survival mechanism favoring PDI expression (Unterberger et al., 2006). Thus, the significant increase of PDI in clinical sheep favors the hypothesis that the IRE1 or ATF6 pro-survival pathways are activated to suppress the proapoptotic action of PERK. This third branch of the UPR directed by ATF6 regulates the transcription of genes that favor survival such as GRP78 and 94 and the protein disulfide isomerase (PDI) proteins (Roller and Maddalo, 2013). It has been mentioned that the inhibition of PERK could be the key to avoid neurodegeneration, it was also reported that the reduction in the expression of BiP/Grp78 accelerates the pathogenesis of the prion *in vivo*, suggesting an important protective role of this chaperone avoiding the spread of infectious prions (Park et al., 2017). Our findings suggest that BiP may play a role in the induction of multiple pathogenic mechanisms involved in neurodegeneration in natural scrapie and that the response to unfolded proteins might be more noticeable in brain areas showing more severe PrP^Sc^ deposition such as the thalamus.

Several authors have focused on the role of protein disulfide isomerase (PDI) in prion diseases. Increased levels of PDI have been observed in brains of patients with sporadic Creutzfeldt-Jakob disease (Yoo et al., 2002) and in murine models of prion disease (Wang et al., 2012; Otero et al., 2021). Yoo et al. (2002) have attributed its upregulation to a cellular defense response against PrP^Sc^ accumulation. Several studies on PDI chaperones in neurodegenerative diseases have provided valuable information about their molecular mechanisms of activation and their protective role against prion neurotoxicity (Hetz et al., 2005; Wang et al., 2012). Dynamic assays of PDI fluctuation in experimental models of scrapie determined that the upregulation of PDI starts at early stages of the disease and persistently increases until later stages (Wang et al., 2012). High levels of Grp58, a member of the PDI family, have been detected in human samples from sCJD patients (Hetz et al., 2005). In our study, as we observed before in models of spontaneous prion disease (Otero et al., 2021), PDI is the ER stress marker that shows the greatest alterations between prion infected and healthy animals (Figure 3). These differences were not only detected between clinical and control animals. Preclinical animals also showed a significant increase of PDI compared to healthy controls in medulla oblongata and hippocampus, which seems to indicate that the levels of this protein start increasing at the early stages of the disease, as reported by other authors (Wang et al., 2012).

During the analysis of the *EIF2AK3*, *HSPA5*, and *P4HB* genes, we observed similar trends for each brain area. Although these trends might be influenced by fluctuations in the housekeeping genes, the analysis of *SDHA* and *GAPDH* showed no differences between each studied brain area (Supplementary Figure S3). Therefore, we believe these trends observed for each brain area are not due to fluctuations in the housekeeping gene expression, but rather to a differential response to ER stress in each brain area, as it has been reported for other markers of neurodegeneration in prion diseases (Makarava et al., 2020; Betancor et al., 2022; García-Martínez et al., 2022).

Moreover, after analyzing the expression of PERK, BiP, and PDI and the corresponding genes that encode each of these proteins (*EIF2AK3*, *HSPA5* and *P4HB*, respectively), we observed certain differences between the gene and protein expression of each of these ER stress markers. These differences between the expression at the protein and gene levels have been previously reported for several proteins, and are typically attributed to other levels of regulation between the transcript and the protein product (Koussounadis et al., 2015). Although gene analyses are widely used to support protein expression analyses, due to these regulation differences that can occur between the mRNA and the protein synthesis, we believe immunohistochemistry reflects in a more proper manner the ER stress phenomena occurring in prion diseases, and therefore, this is the preferred method to analyze these differences throughout our experiment.

Spearman’s test confirmed a strong positive correlation between PDI levels and neuropathological changes. This suggests that the accumulation of PrP^Sc^ aggregates triggers the positive regulation of this stress chaperone perhaps as a neuroprotective response. However, it has also been reported that PDI and GRP58 are involved in apoptosis induced by misfolded proteins (Hoffstrom et al., 2010) and therefore, the increase in PDI observed in this study could be related to the apoptosis produced by the prion disease. In addition, PDI shows a strong positive correlation with the other ER stress markers evaluated. This is not surprising since PDI is an essential activator of PERK (Kranz et al., 2017) and works synergistically with BiP in the correct folding of proteins (Mayer et al., 2000).

As observed with the *HSPA5* gene, encoding BiP, we also observed a significant upregulation of the *P4HB* gene, encoding PDI, in the thalamus region in animals in the preclinical phase. The downregulation of PDI gene in the end-stage of the disease could be related with the development of spongiosis, indicating neuronal loss in scrapie-affected animals, and had been previously observed by Hetz et al. (2005) in the thalamic region.

In this study, we also investigated the possible role of proteasome impairment at different stages of natural prion disease through the analysis of the accumulation of ubiquitin aggregates in the brains of scrapie-infected (clinical and preclinical) and healthy sheep. Several studies in cellular and animal models show that proteasome impairment may be important in the pathogenesis of prion diseases. Impaired proteasome activity and increased levels of ubiquitinated conjugates have been reported in human prion diseases (Ironside et al., 1993) and in the brains of prion-infected mice (Kang et al., 2004; McKinnon et al., 2016), especially in the thalamic area at the early stages of the disease (McKinnon et al., 2016; Otero et al., 2021). However, other authors have not been able to detect alterations in UPS activity *in vivo* (Quaglio et al., 2011). Similar to what we observed with ER stress markers, we detected accumulation of ubiquitin aggregates in the brains of all sheep. However, clinical sheep showed significantly greater accumulation of this protein in numerous brain areas compared to healthy sheep. Differences in ubiquitin accumulation were also detected between the clinical and preclinical group in the hippocampus and basal ganglia (Figure 4), suggesting that ubiquitin accumulation increases as the disease progresses in these animals. Moreover, preclinical animals showed higher accumulation of ubiquitin in medulla oblongata and thalamus, the two brain areas which are earlier affected by prion neurodegeneration, therefore pointing at a relation between prion-related pathogenesis and an increase in ubiquitin accumulation.

Spearman’s correlation test showed that ubiquitin deposition was positively correlated with spongiform lesions and PrP^Sc^ deposits, as well as with ER stress markers BiP and PDI, but not PERK. We may suggest that, in sheep naturally infected with scrapie, as previously described in models of experimental prion disease (Axten, 2017) and in models of spontaneous prion disease (Otero et al., 2021), there is an impairment of the ubiquitin-proteasome system, which is essential in the degradation of misfolded proteins. It has been described that PrP^Sc^ specifically inhibits the β proteolytic subunit of the 26S proteasome (Kristiansen et al., 2007), which can be caused by chronic ER stress since cells under chronic ER stress accumulate non translocated PrP in the cytosol (Orsi et al., 2006). Interestingly, in this study, as we observed with models of spontaneous prion disease, we detected that certain brain areas, such as the thalamus and hypothalamus seem to be affected more frequently by these mechanisms than other brain areas. These differential responses to ER stress and proteasome dysfunction have been reported before (Hetz et al., 2005; Stutzbach et al., 2013).

These differences could be due to the fact that PrP^Sc^ deposits and the response to these protein aggregates are different in each brain region, considering also that the cell population is distinct in each brain area and could influence this response, as has been demonstrated in chronic neuroinflammation involving reactive microgliosis and astrogliosis (Makarava et al., 2020).

Based on the results of this study, the intracellular ubiquitinated deposits detected in preclinical animals could be associated with the early aggregation of PrP^Sc^ since we observed that ubiquitin deposition strongly correlates with spongiosis and PrP^Sc^ accumulation, and UPS activation is indispensable to remove polyubiquitinated substrates and reduce the PrP^Sc^ load (McKinnon et al., 2016).

Although we cannot elucidate that ER stress or UPS dysfunction play a major role in the pathogenesis of scrapie, we have demonstrated that these phenomena are present during the pathogenesis of the disease. However, we should also consider that clinical and preclinical animals in the present study were obtained from different flocks, and it is possible that these sheep were infected with different scrapie strains. Although we observed that ER stress and UPS dysfunction are present in both groups of sheep, it would be interesting to analyze both phenomena throughout the course of the disease in sheep infected with the same scrapie strain and to elucidate whether the alteration in these pathogenic mechanisms varies depending on the infecting prion strain.

Even though, naturally acquired, spontaneous and genetic prion diseases may not share the same pathogenic mechanisms (Quaglio et al., 2011; McKinnon et al., 2016), our results here, in sheep with natural scrapie, are very similar to those observed in spontaneous models of prion disease. However, further biochemical and molecular studies in animals that have developed natural prion disease are necessary to confirm the involvement of ER stress and proteasome malfunction in natural prion disease.

## Data availability statement

The original contributions presented in the study are included in the article/Supplementary material, further inquiries can be directed to the corresponding author.

## Ethics statement

The animal study was reviewed and approved by the Ethical Advisory Commission for animal experimentation of the University of Zaragoza (identification code: P138/15) and performed under their supervision. All procedures involving animals adhered to the guidelines included in the Spanish law for Animal Protection RD53/2013 and the European Union Directive 2010/63 on the protection of animals used for experimental purposes.

## Author contributions

JLO performed most experiments and wrote the original draft of the manuscript. MB gathered the samples, evaluated the neuropathological lesions, and revised the manuscript. SPL collaborated in some of the experiments. RB and JB obtained the funding, supervised the experiments, and revised the final draft of the manuscript. AO obtained the funding, designed the study, supervised the experiments, and revised the final draft of the manuscript. All authors contributed to the article and approved the submitted version.

## Funding

This research was financed by the project n° PID2021-125398OB-I00, funded by MCIN/AEI/10.13039/501100011033/ FEDER and the EU.

## Conflict of interest

The authors declare that the research was conducted in the absence of any commercial or financial relationships that could be construed as a potential conflict of interest.

## Publisher’s note

All claims expressed in this article are solely those of the authors and do not necessarily represent those of their affiliated organizations, or those of the publisher, the editors and the reviewers. Any product that may be evaluated in this article, or claim that may be made by its manufacturer, is not guaranteed or endorsed by the publisher.
