# Supplementary material for: Endoplasmic reticulum stress and ubiquitin-proteasome system impairment in natural scrapie
Source: Front Mol Neurosci. 2023 Apr 21;16:1175364. doi: 10.3389/fnmol.2023.1175364 (PMC10160437; doi:10.3389/fnmol.2023.1175364)
Supplement: Supplementary file 1 [file Data_Sheet_1.PDF]

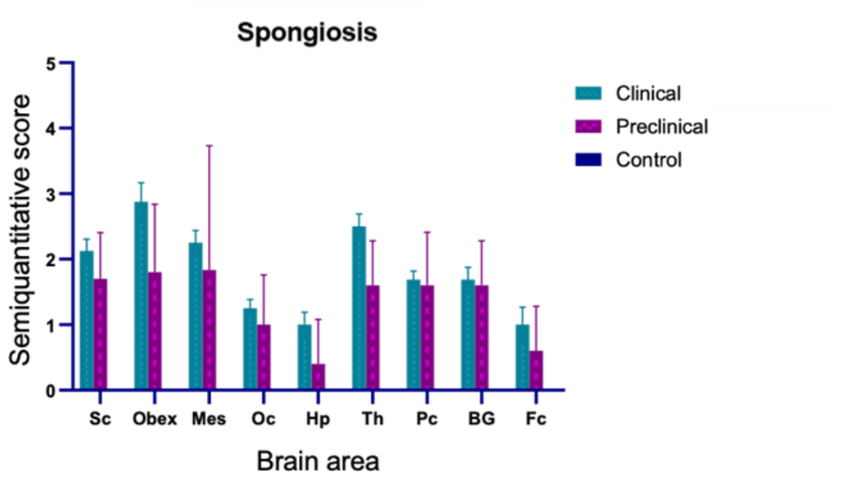

Supplementary Figure 1: Semiquantitative evaluation (from 0=absence of lesions to 5=very intense spongiosis) of the spongiosis found in the clinical (*n* = 8), preclinical (*n* = 5), and control (*n* = 8) sheep used in the present study. No spongiosis was detected in healthy control sheep.

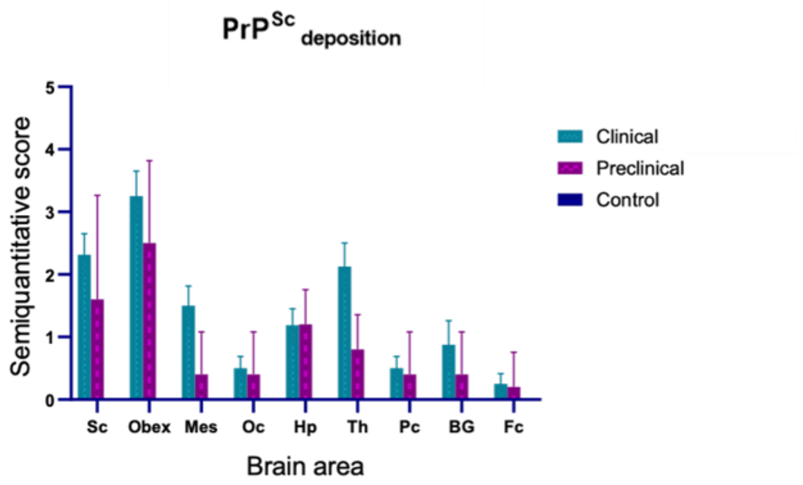

Supplementary figure 2: Semiquantitative evaluation (from 0=absence of PrPSc deposits to 5=very intense PrPSc accumulation) of the PrPSc deposition in the clinical (*n* = 8), preclinical (*n* = 5), and control (*n* = 8) sheep used in the present study. No PrPSc deposition was detected in healthy control sheep.

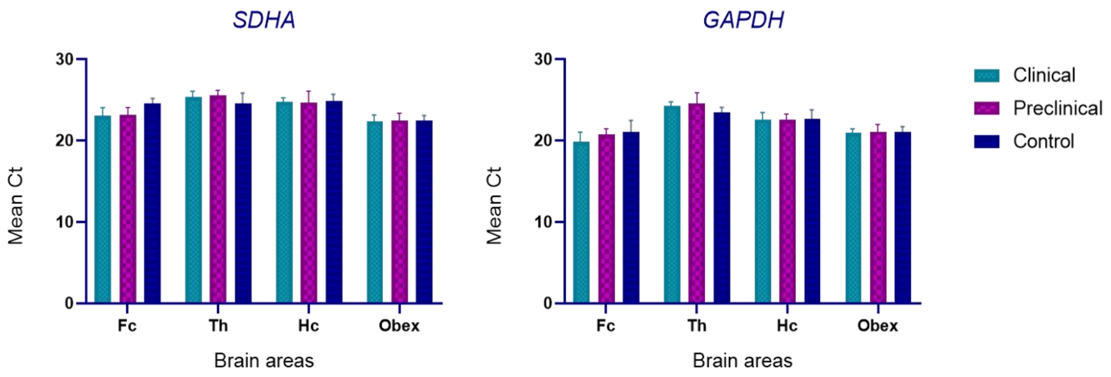

Supplementary Figure 3. Expression of housekeeping genes in different brain areas from the studied sheep. Figure shows the mean Ct of *SDHA* and *GAPDH* housekeeping genes. All brain areas from the different studied groups showed similar mean Ct. No statistical differences were found when comparing groups (clinical *n* = 8, preclinical *n* = 5, and control *n* = 8) using the one-way ANOVA test, followed by the Bonferroni *post hoc* test.
